# Non-invasive modulation reduces repetitive behavior in a rat model through the sensorimotor cortico-striatal circuit

**DOI:** 10.1038/s41398-017-0059-5

**Published:** 2018-01-10

**Authors:** Henriette Edemann-Callesen, Bettina Habelt, Franziska Wieske, Mark Jackson, Niranjan Khadka, Daniele Mattei, Nadine Bernhardt, Andreas Heinz, David Liebetanz, Marom Bikson, Frank Padberg, Ravit Hadar, Michael A. Nitsche, Christine Winter

**Affiliations:** 1 Department of Psychiatry and Psychotherapy, Charité Universitätsmedizin Berlin, Berlin, Germany; 2 Department of Psychiatry and Psychotherapy, Medical Faculty Carl Gustav Carus, Technische Universität Dresden, Dresden, Germany; 3 International Graduate Program Medical Neurosciences, Charité Universitätsmedizin Berlin, Berlin, Germany; 4 Department of Biomedical Engineering, The City College of The City University of New York, New York, NY USA; 5 Cellular Neuroscience, Max-Delbrueck-Center for Molecular Medicine in the Helmholtz Association, Berlin, Germany; 6 Department of Clinical Neurophysiology, University Medical Center, Georg-August-University, Goettingen, Germany; 7 Department of Psychiatry and Psychotherapy, Ludwig Maximillian University, Munich, Germany; 8 Department of Psychology and Neurosciences, Leibniz Research Centre for Working Environment and Human Factors, Dortmund, Germany; 9 Department of Neurology, University Medical Hospital Bergmannsheil, Bochum, Germany

## Abstract

Involuntary movements as seen in repetitive disorders such as Tourette Syndrome (TS) results from cortical hyperexcitability that arise due to striato-thalamo-cortical circuit (STC) imbalance. Transcranial direct current stimulation (tDCS) is a stimulation procedure that changes cortical excitability, yet its relevance in repetitive disorders such as TS remains largely unexplored. Here, we employed the dopamine transporter-overexpressing *(DAT-tg*) rat model to investigate behavioral and neurobiological effects of frontal tDCS. The outcome of tDCS was pathology dependent, as anodal tDCS decreased repetitive behavior in the *DAT-tg* rats yet increased it in wild-type (*wt*) rats. Extensive deep brain stimulation (DBS) application and computational modeling assigned the response in *DAT-tg* rats to the sensorimotor pathway. Neurobiological assessment revealed cortical activity changes and increase in striatal inhibitory properties in the *DAT-tg* rats. Our findings show that tDCS reduces repetitive behavior in the *DAT-tg* rat through modulation of the sensorimotor STC circuit. This sets the stage for further investigating the usage of tDCS in repetitive disorders such as TS.

## Introduction

Repetitive symptoms as observed in among others Tourette Syndrome (TS) can in severe cases hinder social and professional development^1, 2^. The pathology underlying the manifestation of repetitive symptomatology remains inconclusive, yet vast evidence points to an imbalanced striato-thalamo-cortical circuit (STC), where a combined action of dopaminergic hyperresponsivity and striatal disinhibition results in cortical hyperexcitability and ultimately impaired movement control^3–7^.

Current drug therapies, especially those applied for repetitive symptoms seen in TS, lack precision, which may account for their inability to provide sufficient and enduring symptom relief. One treatment strategy allowing for focal intervention is deep brain stimulation (DBS) in which electrical stimulation is delivered directly to pathology-relevant brain areas through implanted electrodes^8^. DBS has already been applied to several brain structures within the STC circuit to reduce repetitive behavior as seen in TS^9–12^. However, despite positive results, its invasive nature hinders a general application and is therefore mostly considered for only severely affected adult patients^13, 14^. To increase the treatment options for repetitive disorders, there is the need for more subtle strategies suitable for a broader patient group.

Transcranial direct current stimulation (tDCS) is a non-invasive, safe and well-tolerated strategy that modulates cortical excitability through application of weak electrical current. The effect on excitability depends on stimulation polarity, with cathodal stimulation decreasing and anodal stimulation increasing membrane excitability at the macroscopic level^15–21^. Beyond acute effects, prolonged stimulation results in neuroplastic after effects, which share some similarities with long-term potentiation and depression^15, 17, 22^. tDCS has already been applied in depression^23^, chronic pain^24^ and schizophrenia^25, 26^ with so far largely positive results. Only one study documents the usage of cathodal tDCS as a mean to reduce cortical hyperexcitability and thus repetitive behavior in TS^27^. Results seem promising, yet a thorough evaluation of its therapeutic relevance in repetitive disorders is missing. Still outstanding is an investigation on the preferable current intensity and stimulation polarity for repetitive pathology and subsequent symptoms. Obviously, such in-depth assessment is clinically challenging, yet is overcome preclinically by employing validated animal models.

The dopamine transporter-overexpressing (*DAT-tg*) rat model exhibits multiple neurobiological abnormalities considered to underlie repetitive disorders, including TS. Apart from far-reaching dopaminergic alterations, these include decrease in striatal GABAergic PV+ expressing interneurons and increased c-fos levels in cortical areas, demonstrating the existence of an imbalanced STC circuit in this model also seen in TS. On a behavioral level, *DAT-tg* rats display amphetamine sensitivity and subsequent repetitive behavior that specifically responds to TS-drug treatment, i.e. administration of the α2 adrenergic and imidazoline receptor agonist clonidine. The occurrence of repetitive behavior in the *DAT-tg* rat is time locked, which allows for evaluation of therapeutic interventions when behavioral manifestation is proven to be most prominent^28^.

In this study, we sought to investigate the effects of frontal tDCS on repetitive behavior in the *DAT-tg* rat. Combining extensive DBS application alongside computational modeling of current spread we sought to identify the sub-circuitry involved in the therapeutic response. Finally, neurobiological assessment of the most therapeutically potent tDCS application enabled mechanistic insight into cortical activity patterns, neurotransmitter levels and inhibitory properties of the striatum. Taken together, our study provides a thorough investigation into the effect of tDCS on repetitive symptomatology and its underlying pathophysiology.

## Materials and methods

### Animals

Experiments were performed in accordance to the European Communities Council Directive of 22 September 2010 (2010/63/EU) after approval by the local ethics committees (Senate of Berlin, Regierungspräsidium Dresden). Experiments were conducted on male Wistar *DAT-tg* rats (*n* = 38) and their respective littermate controls (*wild types* *(wt*) (*n* = 37) with a Sprague Dawley background once they reached postnatal day (PND) >90^28^. Following surgery, animals were single housed in a 12 h light/dark cycle (light on at 06:00 am) with food and water ad libitum. All efforts were made to reduce animal suffering and number of animals used.

### Experimental design

In this study, the experimental groups consisted of subjects randomly allocated to the tDCS or DBS groups, prior to surgeries. Animals ordained to receive tDCS following surgery were subdivided into the tDCS group (*DAT-tg*, *n* = 9,*wt n* = 7) and an overall control group (*DAT-tg*, *n* = 8,*wt n* = 8). Common for all animals was the implementation of an epicranial electrode, surgically fixed onto the scull over the frontal cortex, through which tDCS/sham stimulation was applied. Animals ordained to receive DBS were subdivided into three groups (groups 1–3) prior to surgery and subsequently implanted bilaterally with monopolar electrodes into cortical and subcortical areas of the STC circuit. These included the orbitofrontal cortex (OFC) and caudate putamen (CPu) (group 1) (*DAT-tg*, *n* = 8,*wt n* = 8), the medial prefrontal cortex (mPFC) (group 2) (*DAT-tg*, *n* = 8,*wt n* = 8) or the primary motor cortex (M1) and thalamus (Thal) (group 3) (*DAT-tg*, *n* = 5,*wt n* = 6). All animals recovered for 1 week after surgery before starting experiments. Animals in the tDCS group received either sham, cathodal (100 or 200 µA) or anodal (100, 200 or 300 µA) stimulation. Animals in the DBS group (groups 1–3) received either sham, high (130 Hz) or low (10 Hz) frequency stimulation in the respective brain areas. Animals in the control group only received sham stimulation (see SI, Table S1 for overview of group specifics and number of animals). The repetitive behavioral paradigm described by Hadar et al.^28^ was employed to study the effect of tDCS and DBS on behavior. Stimulation was applied in the beginning of the paradigm and subsequent behavior was assessed during the stereotypy phase. Experiments were conducted in a crossover design and different types of stimulation were applied in a randomized fashion over the course of the experiment. Animals could rest for 1 week in between stimulation. Rats in the control group, receiving sham stimulation, only went through the behavioral paradigm once. These rats served as an overall control group for later neurobiological assessment. For finalization of the experiment, animals were stimulated with the most therapeutic-relevant stimulation settings as assessed by behavioral analysis. As such, animals in the tDCS group received anodal 200 µA stimulation. Following the finalization of the last experimental round, animals were immediately sacrificed and brains were snap frozen for later post mortem neurobiological assessment. Computational modeling was constructed to investigate the electrical current spread mediated by tDCS. The investigators who run the analysis were blind to the group allocation as well as when analyzing the data. More details are included in the supplementary information.

### Surgery

Animals went through surgery after reaching PND 90 (body weight of >280 g). Animals were handled 3–4 days prior to surgery. Surgery was performed under subcutaneous (s.c.) general anesthesia: fentanyl (0.005 mg/kg), midazolam (2 mg/kg) and medetomidine dihydrochloride (0.135 mg/kg). The scull was fixed in a stereotactic frame and bregma was exposed. For animals in the tDCS and control group, an epicranial electrode (2.1 mm diameter) composed of a tubular plastic jacket was placed over the left frontal cortex (AP +3.2; ML1.5) and fixed using glass inomer cement (Ketac Cem; ESPE Dental AG, Seefeld, Germany). For DBS application, monopolar electrodes (0.5 mm, MS303-6-AIU, Plastics One Inc., USA) were implanted bilaterally into the OFC (AP +3.7; ML +2.4; DV –3.3), CPu (AP +1.5; ML +1.5; DV –4.0), mPFC (AP +3.5; ML +0.6; DV –3.4), M1 (AP+ 1.5; ML +2.7; DV –1.5) and Thal (AP –4.1; ML +1.3; DV –6.4). Anchor screws were drilled into the scull for fixation and the individual ground electrode from each DBS electrode was wrapped around the closest screw and fixed with dental cement (Technovit, Heraeus Kulzer GmbH, Wehrheim, Germany). All coordinates were in accordance to Paxinos rat brain atlas^29^. Upon completion of surgery, anesthesia was antagonized by a cocktail of naloxone (0.12 mg/kg), flumazenil (0.2 mg/kg) and antipamzol (0.75 mg/kg). Analgesia (meloxicam: 0.2 mg/kg, s.c.) was given for 3 days following surgery.

### Repetitive behavior paradigm

As identified by Hadar et al.,^28^ *DAT-tg* rats display a time-locked induction of repetitive behavior following the injection of amphetamine. In this experiment, animals were injected with amphetamine (2.0 mg/kg, i.p., dissolved in 0.9% saline at a volume of 1.0 ml/kg, Sigma Aldrich, Germany) and thereafter immediately subjected to stimulation (either tDCS or DBS). Animals in the DBS group received 60 min of stimulation. Cables were removed following DBS application and the animals could move freely for additional 60 min. Animals in the tDCS conditions received 30 min of tDCS or sham stimulation, respectively. Cables and jackets were removed following tDCS application and the animals could move freely for an additional 90 min (See SI, Figure S1). Behavior was recorded via web cameras throughout the paradigm. The occurrence of repetitive behavior (oral stereotopy or head movements) was later analyzed during the stereotopy phase (90–120 min following injection) by a blinded observer using the scoring protocol of Hadar et al.^28^

#### tDCS application

For delivery of tDCS, the epicranial electrode was filled with saline (0.9%) (contact area of 3.5 cm^2^) after which a gold pin was inserted for stimulation application. A counter electrode (8 cm^2^; From Physiomed Elektromedizin AG, Schnaittach, Germany) was placed onto the thorax together with electroencephalography (EEG) conducting crème (GVB-geliMED KG, Germany) and kept in place by a jacket^30, 31^. Animals were exposed to either 30 min anodal (100, 200 or 300 µA), cathodal (100 or 200 µA) or sham stimulation in the beginning of the repetitive behavioral paradigm. Both cathodal and anodal stimulation were applied by a computer-interfaced current generator (STG4008 Multi Channel System GmbH Reutlingen, Germany). The current strength was ramped for 10 s to prevent abrupt interruption and stimulation break effects. For sham stimulation, animals were connected to the system, yet no current was flowing.

#### DBS application

DBS was applied as biphasic 100 μs pulses with either a current intensity of 150 μA and frequency of 130 Hz (high frequency) or with a current intensity of 300 μA and frequency of 10 Hz (low frequency). Stimulation was controlled by the STG4008 Multi Channel System GmbH Reutlingen, Germany. At 1 day prior to testing, DBS or sham stimulation was performed twice for 1 h (morning and afternoon). On testing day, animals were subjected to 60 min of either high- or low-frequency stimulation in the respective areas during the beginning of the behavioral paradigm. Sham stimulation was applied in an identical fashion yet no current was flowing.

### Post mortem neurobiological assessment

#### Decapitation and snap freeze

Animals were immediately sacrificed following finalization of the experiment. Brains were extracted within less than 20 s, snap frozen for 2 min in methylbutane cooled with liquid nitrogen to a temperature of −40 °C and then stored at −80 °C until required. Next to electrode localization, frozen coronal sections of 1 or 0.5 mm were cut on a cryostate (see Table S2 for coordinates).

#### High-performance liquid chromatography (HPLC)

Tissue samples were taken via micropunches of 1 mm diameter and were homogenized by ultrasonication in 250 µl (per punch) 0.1 N perchloric acid at 4 °C. Then, 100 µl of the homogenate was added to equal volumes of 1N sodium hydroxide for measurement of protein content. The remaining homogenate was centrifuged at 13,000 g and 4 °C for 15 min. The supernatant was added to equal volumes (20 µl) of 0.5 M borate buffer and stored at −80 °C for subsequent analyses of amino acids. The remaining supernatant was used for immediate measurement of monoamines. Monoamine levels (3,4-dihydroxyphenylacetic acid (DOPAC) and dopamine (DA)) were measured by HPLC with electrochemical detection as previously described^32, 33^.

#### Quantitative polymerase chain reaction (qPCR)

Tissue samples from the left hemisphere were taken via micropunches of 1 mm diameter from the mPFC, M1 and OFC. Further tissue samples were taken in the same way from both hemispheres from the CPu. Tissue was homogenized by ultrasonication in the buffer provided by the NucleoSpin RNA/Protein-Kit (Machery-Nagel, Düven, Germany). The total RNA and protein was extracted as recommended in its user manual. RNA concentrations were determined using a Nanodrop Spectrophotometer (peqlab). cDNA was synthesized using the High Capacity RNA-to-cDNA Kit (Lifetechnologies). TaqMan qPCR was performed with StepOne Real-Time PCR System (Lifetechnologies) using TaqMan fast advanced master mix (Lifetechnologies). The following TaqMan Gene Expression assays (Lifetechnologies) were used: Pvalb Assay (Rn00574541_m1) and c-Fos Assay (Mm00487425_m1). CT values were normalized to the housekeeping gene GFAP (Rn01253033_m1). Fold change was calculated using the ∆∆CT method.

#### Electrode localization

At the respective coordinates, brains were sliced into 20 mm coronal sections and Nissl-stained for light microscopic inspection of electrode tip placements as previously explained^34–36^. One animal with a wrong-positioned electrode was excluded from the study.

### Computational modeling

To determine the effect of various current densities on the cortex, a state-of-the-art model was constructed from a magnetic resonance imaging (MRI; 7.0 Tesla70/30 Bruker Biospec) and micro computed tomography scan (Siemens Inveon) of a template rat head^37^.

#### MRI data collection and segmentation

MRI resolution was 0.282 mm, as previously mentioned^37^. The scans were segmented into 9 tissues: skin, skull, cerebral spinal fluid (CSF), air, gray matter, white matter, hippocampus, cerebellum and spinal cord. A Rat Brain Atlas^38^ was used to identify the hippocampal region of the brain. Remaining brain regions were appropriately grouped as either gray or white matter. Manual segmentation was used to generate an initial segmentation of scalp, skin, CSF, air, gray matter, white matter, hippocampus, cerebellum and spinal cord. Tissue continuity was verified after segmentation by extensively reviewing the data. Further manual adjustments were made to guarantee continuity and improve the segmentation accuracy to closely match the tissue masks to the real anatomy of the rodent using ScapIP 7.0 (Simpleware Ltd, Exeter, UK).

#### Modeling of tDCS

The tDCS in vivo electrode placement protocol described above was modeled in SolidWorks (Dassault Sysemes Corp. Waltham, MA) and imported into ScanIP for meshing. The modeled epicranial electrode had a contact area of 3.5 cm^2^ and was placed in accordance to coordinates used in the behavioral experiment (AP: +3.2; ML:1.5). The 1.0 mm diameter gold pin serving as the anode was placed on the skull inside of the epicranial electrode. An 8 cm^2^ cathode was placed on the thorax with EEG conducting crème as an electrolyte. An adaptive tetrahedral meshing algorithm was used in ScanIP to generate meshes of 8 × 10^6^ quadratic elements. A Finite Element Method (FEM) model was created in COMSOL Multiphysics 4.3 (COMSOL, Inc., Burlington, MA) using the mesh mentioned above. The model was created using electrostatic volume conductor physics with material conductivities defined as follows (in S/m): skin, 0.465; skull, 0.01; CSF, 1.65; air, 1e^−15^; spinal cord, 0.126; gray matter, 0.276; white matter, 0.126; hippocampus, 0.126; cerebellum, 0.276; dental cement, 1e^−15^; electrode jacket, 1e^−15^; saline, 1.4; and electrode, 5.99e^7^. Conductivity values were taken from a combination of in vitro and in vivo measurements^39, 40^. Current boundaries were applied to simulate direct current stimulation, and internal boundaries between tissues were assigned the continuity condition (n _*_ (J_1_−J_2_) = 0), and the Laplace equation (∇ ∗ (σ∇V) = 0) was solved. The surface of the cathode was grounded (V = 0) while the surface of the anode had a current density of 3.252e^−4^ A/m^2^. All other exterior surfaces were electrically insulated. Brain current density data were collected from the left cortical hemisphere above the corpus callosum and averaged for analysis. High-resolution models predicted the concentration and distribution of brain current density for the in vivo rodent model using the electrode montage.

### Statistics

Sample size was chosen based on the convention that for behavioral experiments *n* of 8–10 ensures adequate power to detect a prespecified effect size and on our previous experience with the chosen methods^28^. Inclusion criterion was the complete endurance of the neuromodulation period. This criterion is an integral part of the study objectives and design as it was designed to study DBS as a preventive avenue. Exclusion criterion for outliers was +/−2 standard deviations of the means. Behavioral analysis was performed using a one-way analysis of variance (ANOVA) repeated measure with Treatment as variables. Post-hoc tests utilized the Holm–Sidak for multiple comparisons. Neurobiological analysis for HPLC was performed using a two-Way ANOVA with treatment (sham, tDCS and DBS) and phenotype (*DAT*-*tg* vs. *wt*) as variables. Analysis of qPCR data was conducted following normalization to sham stimulation, with a one-way ANOVA used for c-fos analysis and a non-parametric Mann–Whitney test employed for parvalbumin (PV) analysis. Statistical significance was set at *p* < 0.05. Results are expressed as mean ± s.e.m. The experiment shown was replicated once in our lab.

## Results

### Behavioral effects

**Fig. 1:**
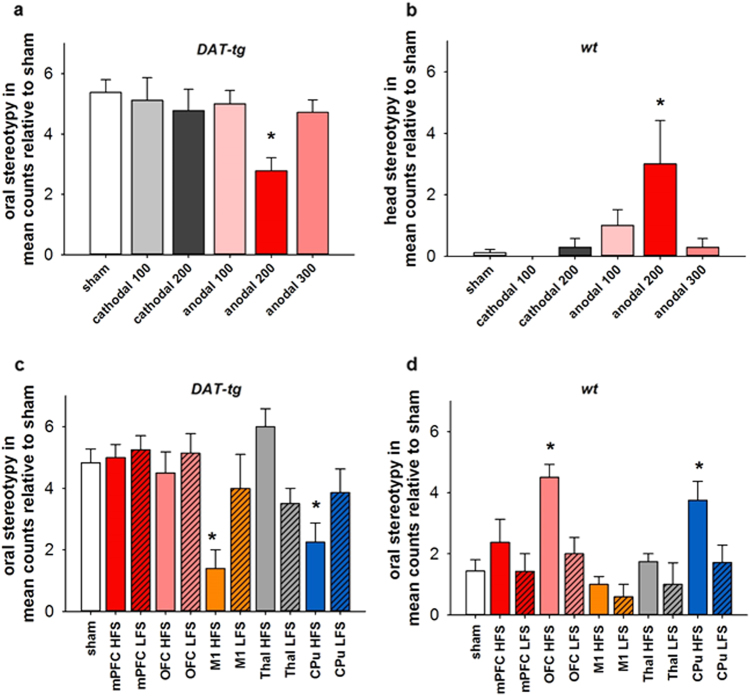
The effects of tDCS and DBS on repetitive behavior **a** tDCS (anodal/cathodal at 100 , 200  or 300  µA) effects on the occurrence of oral stereotypy in the *DAT-tg* rats in relation to sham stimulation. **b** Effect of tDCS (anodal/cathodal at 100 , 200 or 300  µA) on the occurrence of head movements in the *wt* rats in relation to sham stimulation. **c** Low- and high-frequency DBS (mPFC, OFC, M1, Thal and CPu) effects on the occurrence of oral stereotypy in the *DAT-tg* rats in relation to sham stimulation. **d** Low- and high-frequency DBS (mPFC, OFC, M1, Thal and CPu) effects on the occurrence of oral stereotypy in the *wt* rats in relation to sham. *wt* wild-type rats, *DAT-tg* dopamine transporter-overexpressing rats, mPFC medial prefrontal cortex, OFC orbitofrontal cortex, M1 primary motor cortex, Thal thalamus, CPu caudate putamen. All data are given as mean ± s.e.m. Asterisks (*) indicate significant differences between stimulation conditions with *p* < 0.05

The after-effect of tDCS (anodal and cathodal) on behavior was assessed during the stereotypy phase of a repetitive paradigm^28^. In *DAT-tg* rats, one-way repeated measure analysis of variance (rmANOVA) tDCS effects on behavior in relation to sham revealed a significant effect for treatment (F^5,33^ = 2.727, *p* = 0.036), with a further post-hoc test showing that frontal anodal tDCS at 200 µA significantly reduced oral stereotypy when compared to sham stimulation (*p* = 0.012) (Fig. 1a). In *wt* rats, one-way rmANOVA showed a significant effect for the factor treatment (F^5,28^ = 3.388, *p* = 0.016), with anodal tDCS at 200 µA significantly increasing head movements in comparison to sham stimulation (*p* = 0.015) (Fig. 1b), whereas no effect of either anodal or cathodal tDCS was seen on oral stereotypy (data not shown). The effect of high- and low-frequency DBS was assessed following the application to several cortical and subcortical structures. In *DAT-tg* rats, one-way rmANOVA showed a significant effect for treatment (F^10,51^ = 4.112, *p* < 0.001), as oral stereotypy significantly decreased following high-frequency DBS to the CPu (*p* = 0.001) and M1 (*p* = 0.019) in comparison to sham stimulation. In contrast, no effect was found following DBS to the mPFC, OFC or thalamus (thal) at either low- or high-frequency stimulation (Fig. 1c). In *wt* rats, a significant effect was found for the factor treatment (F^10,54^ = 4.102, *p* < 0.001), with an increase in oral stereotypy seen after high-frequency DBS to the OFC (*p* = 0.001) and CPu (*p* = 0.026) in comparison to sham stimulation. No effect was seen following DBS to the mPFC, M1 or thal at either low- or high-frequency stimulation (Fig. 1d).

### Activity measures

**Fig. 2:**
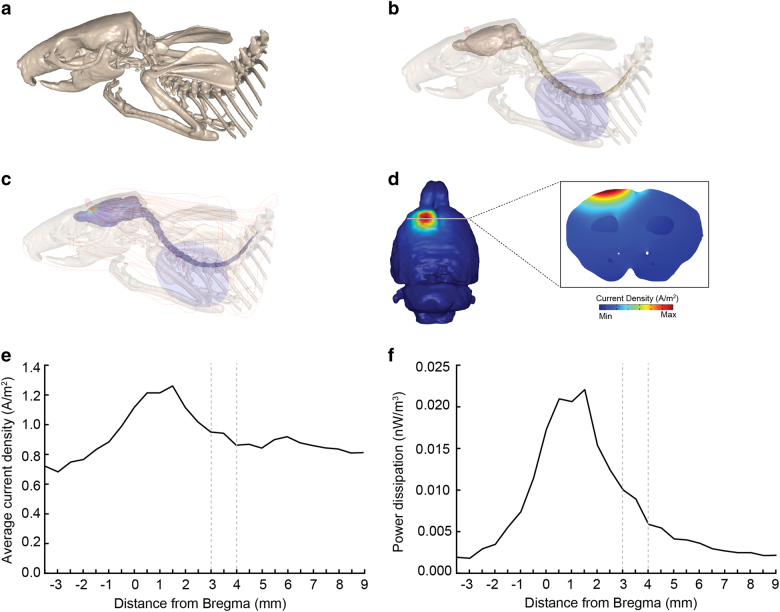
A realistic high-resolution MRI-based rat computational model and predicted average current density and power dissipation **a** Illustration of render of bone generated from images and segmented for the FEM model. **b** Electrode positioning on the entire model with transparent skeleton, brain, spines and electrodes. The red pellet-shaped anode electrode and blue cathode electrode are positioned as aforementioned. **c** Uniformly seeded streamlines from the top surface of the anode electrode that were proportional to the logarithm of current density magnitude. Trajectories of the streamlines predict direct current flow across different brain tissues. **d** Predicted current density at the surface of the brain and a slice view of the distribution showing peak current density during tDCS. Lowest brain current density values are represented in blue, and greater brain current density values are represented in dark red. **e** Average current density across the cortex following anodal tDCS. **f** Average power dissipation across the cortex following anodal tDCS. The dotted lines in both **e**, **f** illustrate the boundary of the electrode in accordance to bregma

**Fig. 3:**
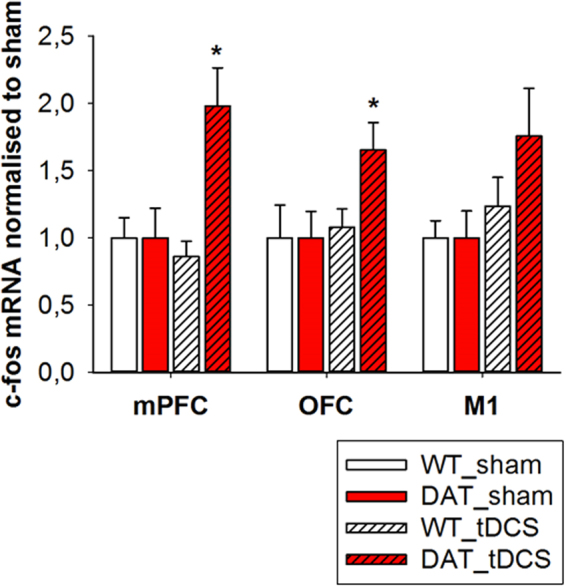
C-fos expression levels Figure showing c-fos mRNA expression levels in the mPFC, OFC and M1 in the *DAT-tg* and *wt* rats following tDCS (anodal, 200  µA) in relation to sham. *wt* wild-type rats, *DAT-tg* dopamine transporter-overexpressing rat, OFC orbitofrontal cortex, M1 primary motor cortex, tDCS transcranial direct current stimulation. All data are given as mean ± s.e.m. Asterisks (*) indicate significant difference between stimulation with *p* < 0.05

The current density resulting from anodal tDCS at 200 µA was predicted by computational modeling. Current density distribution following anodal tDCS for the in vivo rodent model is shown in Fig. 2c. Results show a prominent peak of both average current density and average power dissipation approximately 1.5 mm anterior to bregma (average current density 1,18825E–06; average power dissipation 2,0979E–11) (Fig. 2a, b). Analysis of c-fos mRNA expression levels was conducted to quantify cortical activity pattern following effective tDCS in relation to sham stimulation. A one-way ANOVA showed a significant difference in the mPFC, (F^1,13^ = 7.732, *p* = 0.016) and OFC (F^1,10^ = 5.129, *p* = 0.043) such that within these areas anodal tDCS at 200 µA significantly increased c-fos mRNA levels in the DAT-tg rats. No difference in c-fos levels was detected in the *wt* rats. (Fig. 3).

### Neurobiological assessment

**Fig. 4:**
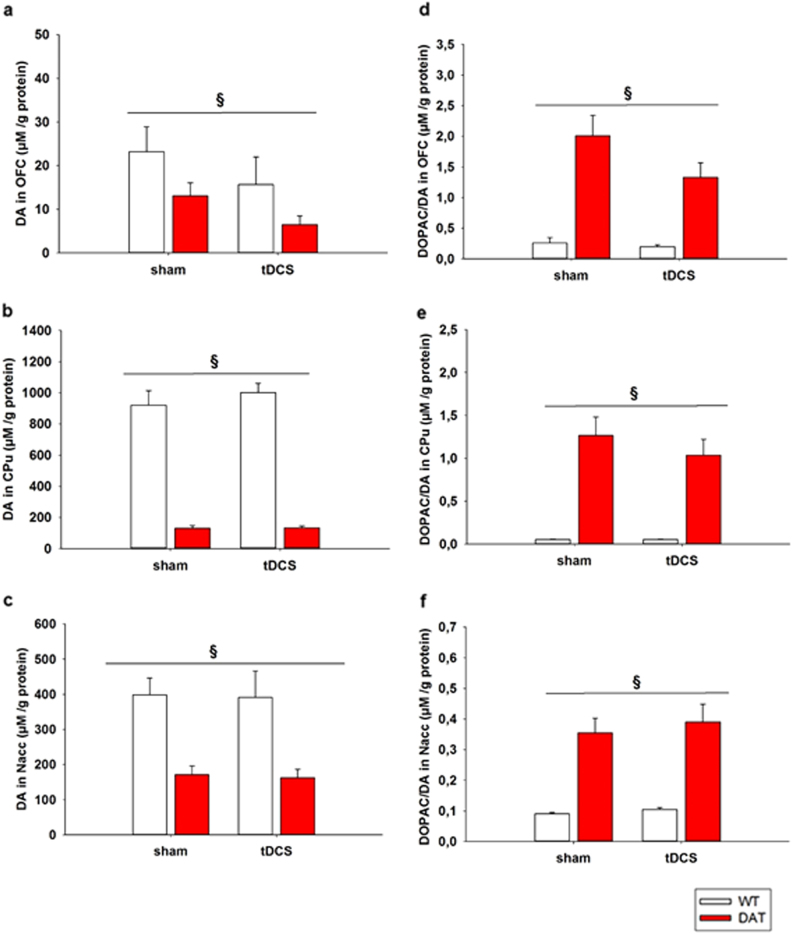
Dopamine levels Figure showing dopamine content (µM/g protein) in the **a** OFC **b** CPu and **c** NAcc following sham and tDCS (anodal, 200 µA) and dopamine turnover (DOPAC/DA) (µM/g protein) in the **d** OFC **e** CPu and **f** NAcc following sham and tDCS (anodal, 200 µA). *wt*: wild-type rats, *DAT-tg* dopamine transporter-overexpressing rat, DA dopamine, DOPAC 3,4-Dihydroxyphenylacetic acid, OFC orbitofrontal cortex, CPu caudate putamen, NAcc nucleus accumbens, tDCS transcranial direct current stimulation. All data are given as mean ± s.e.m. Asterisks (*) indicate significant difference between stimulation protocols, and the symbol (§) indicates significant differences between phenotype with *p* < 0.05

Neurobiological investigations were conducted for frontal anodal tDCS at 200 µA. HPLC post mortem biochemical analysis was made to investigate the persisting effect on neurotransmitter levels. Based on previously proven relevance^28^, DA levels and DA turnover (DOPAC/DA) were assessed in the OFC, CPu and NAcc. Looking at DA levels, two-way ANOVA revealed a significant main effect for phenotype in the OFC (F^1,22^ = 5.270, *p* = 0.032), CPu (F^1,23^ = 247.623, *p* < 0.001) and NAcc (F^1,23^ = 29.285 *p* < 0.001) with *DAT-tg* rats generally displaying lower DA levels in comparison to *wt* rats (Fig. 4a–c, Table 1). Further investigation into DA turnover revealed in the OFC, CPu and NAcc a significant effect for phenotype (OFC: F^1,22^ = 37.471, *p* < 0.001; CPu: F^1,23^ = 43.789, *p* < 0.001; NAcc: F^1,22^ = 45.293, *p* < 0.001), with DAT-*tg* rats showing a higher degree of DA turnover as compared to the *wt* rats (Fig. 4d–f, Table 1).

**Table 1:** Neurotransmitter contents

Region	Transmitter	Pheno	Stim	µM/g protein	Two-way ANOVA	DF	*F*-value	*P*-value
	DA	wt	sham	23.185±4.319	Pheno	(1,22)	5.27	0.032^a^
OFC			tDCS	15.630±4.731	Stim	(1,22)	2.82	0.107
		DAT-tg	sham	13.034±3.740	Interaction	(1,22)	0.0129	0.911
			tDCS	6.435±3.999				
	DOPAC	wt	sham	0.257±0.266	Pheno	(1,21)	37.471	<0.001^a^
			tDCS	0.194±0.291	Stim	(1,21)	4.243	0.052
		DAT-tg	sham	2.353±0.230	Interaction	(1,21)	3.328	0.082
			tDCS	1.328±0.266				
	DA	wt	sham	963.86±52.35	Pheno	(1,23)	247.623	<0.001^a^
CPu			tDCS	1002.0±61.95	Stim	(1,23)	0.146	0.706
		DAT-tg	sham	129.65±48.97	Interaction	(1,23)	0.105	0.749
			tDCS	132.78±52.35				
	DOPAC	wt	sham	0.052±0.160	Pheno	(1,23)	43.789	<0.001^a^
			tDCS	0.052±0.190	Stim	(1,23)	0.497	0.497
		DAT-tg	sham	1.264±0.150	Interaction	(1,23)	0.495	0.495
			tDCS	1.035±0.160				
	DA	wt	sham	398.19±40.73	Pheno	(1,23)	29.285	<0.001^a^
Nacc			tDCS	390.92±48.19	Stim	(1,23)	0.0359	0.851
		DAT-tg	sham	171.01±38.11	Interaction	(1,23)	0.00028	0.987
			tDCS	162.32±40.74				
	DOPAC	wt	sham	0.090±0.038	Pheno	(1,22)	45.293	<0.001^a^
			tDCS	0.104±0.045	Stim	(1,22)	0.372	0.548
		DAT-tg	sham	0.354±0.036	Interaction	(1,22)	0.072	0.791
			tDCS	0.390±0.041				

*OFC*orbitofrontal cortex, *NAcc* nucleus accumbens

Neurochemical content was examined in the *DAT-tg* and *wt* rats following sham and tDCS. Dopamine levels and dopamine turnover were measured in the medial OFC, NAcc and CPu. Data are presented as mean ± s.e.m. Asterisks (*) indicate significant differences between phenotype with *p* < 0.05

**Fig. 5:**
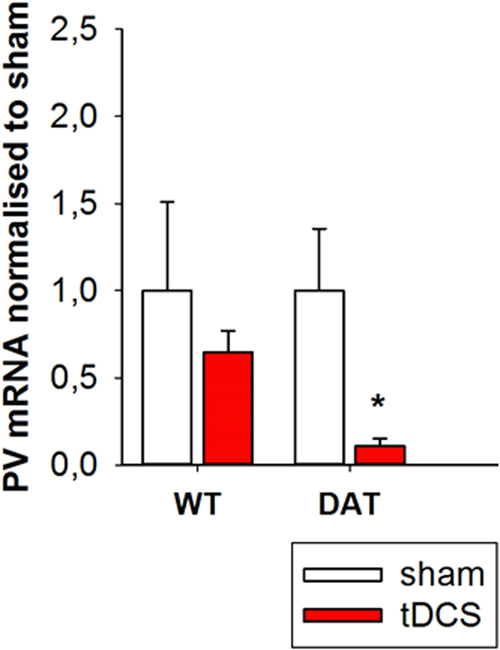
Parvalbumin mRNA levels in the caudate putamen Figure showing the Pv+ mRNA expression levels in CPu in the *DAT-tg* and *wt* rats following tDCS (anodal, 200 µA) in relation to *wt* sham. *wt* wild-type rats, *DAT-tg* dopamine transporter-overexpressing rat, Pv+ parvalbumin, CPu caudate putamen, tDCS transcranial direct current stimulation, M1 primary motor cortex. All data are given as mean ± s.e.m. Asterisks (*) indicate significant differences between stimulation protocols with *p* < 0.05

To quantify inhibitory properties of the striatum, a qPCR on PV mRNA in the CPu was employed. The Mann–Whitney test revealed a significant reduction in PV mRNA levels following tDCS in the *DAT-tg* rats as compared to sham stimulation (Mann–Whitney *U* = 1.500, *p* = 0.005). No difference was observed in the *wt* rats. (Fig. 5). See Supplementary Information (SI) for neurobiological assessment and computational modeling of DBS application.

## Discussion

Here, we demonstrate that anodal tDCS applied at 200 µA to the rat frontal cortex significantly reduced repetitive behavior in the DAT-*tg* rat model. By mathematically modeling current density distribution and multisite DBS application, we further show that the tDCS-therapeutic effects involved a modulation of the sensorimotor STC circuit.

The cumulative outcome of tDCS relies on several factors including stimulation intensity, duration and polarity in combination with the initial neuronal baseline activity, neurotransmitter profile and target structure composition^41–43^. These interacting components all challenge direct comparisons between studies and demonstrate the need for specific assessment of tDCS effects in a given disorder. Well-controlled studies in appropriate animal models provide a platform for investigating interactions between the respective pathology and stimulation parameters that ultimately will determine the tDCS procedure needed for therapeutic relief. We found repetitive behavior to significantly decrease in *DAT-tg* rats following application of frontal anodal tDCS (200 µA), whereas the same stimulation type increased it in *wt* rats. Higher (300 µA) and lower (100 µA) current intensities had no effect in either phenotype. In the context of repetitive behavior, these results demonstrate a polarity-specific and non-linear dose dependency of tDCS. *DAT-tg* rats display heightened cortical activity levels due to an underlying hyperresponsive dopaminergic system in comparison to *wt* rats^28^. In line with this, it was previously shown that alterations in the underlying dopaminergic state, either pharmacologically induced or due to disease-related alterations, could modify and even invert the facilitating effects of tDCS^44–48^. As the final tDCS response depends on initial cortical activity and underlying DA levels, the opposing behavioral outcome between the two phenotypes further reflects a state-dependent modulation of tDCS. Of note, repetitive behavior observed in *wt* rats (i.e., head movements) following tDCS differed from that induced in *DAT-tg* rats (oral stereotypy) further corroborating the notion of a differential modulation mediated by tDCS in the two phenotypes.

The STC circuit is composed of several topographically organized pathways that are separately associated with different aspects of repetitive symptomatology. The sensorimotor circuit is considered the site of tic origin, whereas the limbic and associative circuits are linked to comorbidity and cognitive deficiency^49, 50^. To further identify the sub-circuit involved in modulation of repetitive behavior in *DAT*-*tg* rats by tDCS, we applied DBS to several cortical and subcortical areas of the STC circuit. On a cortical level, repetitive behavior significantly decreased in *DAT-tg rats* when stimulating the primary motor cortex (M1). Contrary, repetitive behavior in *DAT-tg* rats was not affected when DBS was applied to the mPFC or OFC. This indicates that modulation of sensorimotor pathways is essential for improving movement control in *DAT-tg* rats, a notion that is further corroborated by our findings showing that also DBS of the CPu but not the Thal decreased repetitive behavior in *DAT-tg* rats. Of note, DBS modeling identified maximum current density to subside especially within the M1 and CPu following high-frequency DBS, indicating elevated susceptibility of these regions to stimulation (see SI, Figure S5). High-frequency DBS has, as opposed to low-frequency DBS and sham stimulation, proven capable of modulating widespread neuronal circuits, which subsequently has been linked to its therapeutic effect^51^. In correlation, repetitive behavior only decreased in DAT-*tg* rats following high-frequency DBS, whereas the effect of low-frequency DBS was equivalent to sham stimulation.

Indeed, tic generation has been often linked to abnormal motor cortex excitability, with a subsequent modulation of the M1 needed for therapeutic relief^27, 52–58^. Frontal anodal tDCS applied in this study ultimately targets multiple cortical areas, which hinders the ability to specify the precise cortical region underlying the therapeutic response. To further dissect cortical impact of anodal tDCS, an individualized model of current distribution was constructed. Despite uniform application, tDCS has shown to produce a complex spatial pattern of current density across cortical regions^42^. Indeed, variation in current density was found across the cortex, with results revealing a specific peak of average current density and power dissipation over the coordinates matching the M1 target, of which DBS exerted beneficial effects. Together, these finding identify the motor cortex as a potential key region for the therapeutic action of anodal tDCS.

Nevertheless, our data also show that M1 is not the only cortical structure of relevance to repetitive behavior in general and tics in particular but that both the OFC and the mPFC also play a crucial role. Same as frontal anodal tDCS, DBS applied to the OFC led to repetitive behavior in *wt* rats, which underlines the involvement of the OFC in the occurrence of repetitive behavior^59–61^. The mechanism of action leading to this stimulation-induced behavior in *wt* rats still remains to be elucidated. Further, our data on c-Fos mRNA showed persistent activity in the OFC and mPFC in *DAT-tg* rats after tDCS, whereas no change was observed in the *wt* rats. A major proportion of TS patients ultimately gain control over symptoms when approaching adulthood^62^. The ability to voluntarily suppress tics has been linked to adaptive cortical changes, by which a persistent, increased activity between frontal and sensorimotor areas ultimately minimizes unwanted movement execution. As opposed to healthy subjects, this adaptive cortical interaction is continuously heightened in TS and persists even during voluntary movement suppression^3, 54^. In correlation, anodal tDCS has been shown to induce compensatory cortical activity changes in Parkinson’s disease, which has been linked to the symptom relief mediated by tDCS^63, 64^. The persistent cortical activity observed after the end of anodal tDCS in the *DAT-tg* rat leaves thought for further investigation into how cortico–cortical interaction between the frontal and sensorimotor cortices are modified by tDCS and thus ultimately influences behavior in the *DAT-tg* rats.

Cortical hyperexcitability is considered a consequence of underlying striatal disinhibition. In this regard, we observed that CPu-DBS reduces repetitive behavior in *DAT-tg* rats and increases it in *wt* rats. DBS is regarded as being capable to induce functional inhibition of the stimulated target, however preclinical studies show that DBS effects are phenotype dependent and thus rely on the underlying pathology^34, 65–67^. Hence, the induction of repetitive behavior in *wt* rats following CPu-DBS may reflect striatal silencing shown to drive repetitive behavior, whereas the reduction in the *DAT-tg* rats indicates the need for modifying the dysfunctional striatum to improve symptoms.

Striatal disinhibition in TS is largely believed to originate from a loss of GABAergic PV+ expression interneurons^68, 69^. This notion is further corroborated by animal studies showing that repetitive behavior occurs in both rodents and primates following striatal lesion but also selective GABAergic pharmacological inactivation of the striatum^70–72^. Identical to TS patients, *DAT-tg* rats display a specific reduction of striatal PV+ expressing interneurons^28^. In accordance to the translational importance of striatal disinhibiton in both TS patients and *DAT-tg* rats, we observed a general decrease in PV+ mRNA levels in the striatum following anodal tDCS. Interestingly, PV+ mRNA expression levels and loss of PV+ interneurons promote opposing effects on activity balance, as decrease in mRNA levels enhances whereas interneuron loss reduces inhibition, respectively^73^. In accordance, studies investigating the role of PV+ in synaptic transmission show that decrease in PV+ expression levels increase short-term facilitation of GABA release, thus leading to increased inhibition^73, 74^. This suggests that modulation of striatal activity properties is involved in the reduction of repetitive behavior in the *DAT-tg* rats. However, more studies are needed on this matter including the investigation into how tDCS affects mRNA turnover levels and how this translates into expression of PV+ striatal interneurons. Of note, a significant decrease in striatal PV mRNa levels was also observed following application of therapeutic M1-DBS in the *DAT-tg* rats, indicating a general proficiency of M1 stimulation to affect the CPu (see SI, Figure S4). Moreover, it has been shown that only anodal tDCS but not cathodal stimulation of the M1 can modulate subcortical structures of the STC circuit^74^. Given that we did not observe improvement in behavior following cathodal stimulation at any intensity tested, we may speculate that the therapeutic effect of anodal tDCS at least in part depends on its ability to affect striatal inhibition reflected in abnormal PV+ control.

The majority of literature supports the hypothesis of a deregulated DA system in TS pathology. This notion is supported by clinical findings as DA antagonists ameliorate and DA stimulants exacerbate tics, respectively.^75, 76^ In line, *DAT-tg* rats display a general decrease in DA levels and increase in DA turnover due to DAT overexpression. Our results show no effect of tDCS on either DA levels or turnover, indicating that the therapeutic effect of tDCS goes beyond the dopaminergic system. Of note, therapeutic DBS led to modulation of the dopaminergic system in the *DAT-tg* rats, which correlates with the mechanism of DBS in TS patients (see SI, Figs. S2–S3 and Table S3)^77^.

Taken together, we find that tDCS reduces repetitive behavior in the *DAT-tg* rats presumably through a restoration of the previously imbalanced sensorimotor STC circuit. Given the importance of the STC circuit in repetitive pathology, this indicates that tDCS may be employed as stimulation approach to provide symptom relief for repetitive disorders. From a clinical perspective, the primary motor cortex is the best-studied brain area with respect to tDCS application. Based on the initial contradicting line of reasoning, anodal tDCS to the motor cortex has so far not been assessed as a mean to *reduce* cortical excitability for treatment of, for example, TS. Yet, given the underlying pathology found in repetitive disorders and in TS, application of anodal tDCS might just have a positive effect on repetitive behavior as indicated by our findings. Our results thus set the stage for further investigation into the therapeutic application of tDCS in repetitive disorders. If successful, tDCS would provide a non-invasive and safe treatment strategy suitable for patients at all age groups.

## Electronic supplementary material

Supplementary information
