# Supplementary material for: Non-invasive modulation reduces repetitive behavior in a rat model through the sensorimotor cortico-striatal circuit
Source: Transl Psychiatry. 2018 Jan 10;8:11. doi: 10.1038/s41398-017-0059-5 (PMC5802458; doi:10.1038/s41398-017-0059-5)
Supplement: Supplementary file 1 — Supplementary information [file 41398_2017_59_MOESM1_ESM.docx]

**Supplementary information**

**Non-invasive modulation reduces repetitive behavior in a rat model through the sensorimotor cortico-striatal circuit**

Henriette Edemann-Callesen, Bettina Habelt, Franziska Wieske, Mark Jackson, Niranjan Khadka, Daniele Mattei, Nadine Bernhardt, Andreas Heinz, David Liebetanz, Marom Bikson, Frank Padberg Ravit Hadar, Michael A. Nitsche, Christine Winter*

1. **Experimental setup**


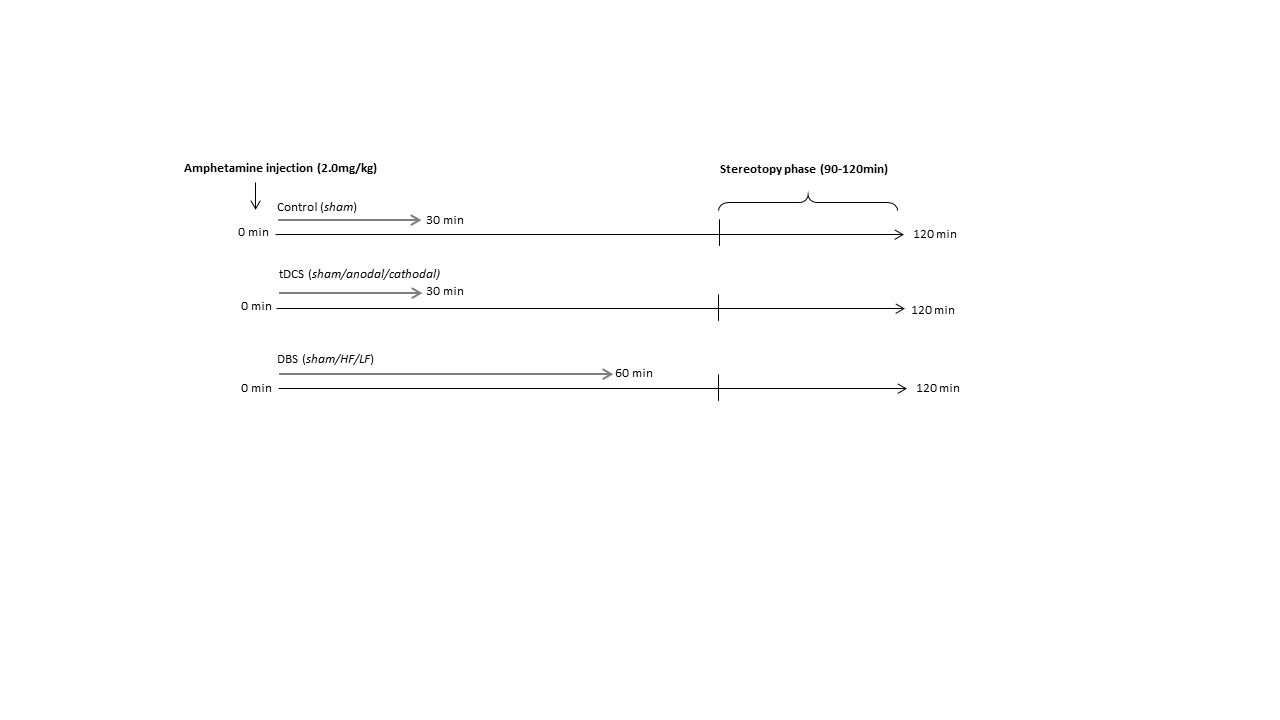


**Figure S1: Repetitive behavioral paradigm.** A timeline of the repetitive behavioral paradigm starting from injection of amphetamine (0 min) (2.0mg/kg) to the occurrence of repetitive behavior (90-120min, stereotypy phase). Stimulation (sham, tDCS and DBS) was applied in the beginning of the paradigm. Behavioral effects were assessed in the stereotypy phase. tDCS: transcranial direct current stimulation; DBS; deep brain stimulation, HF: high frequency stimulation, LF: low frequency stimulation


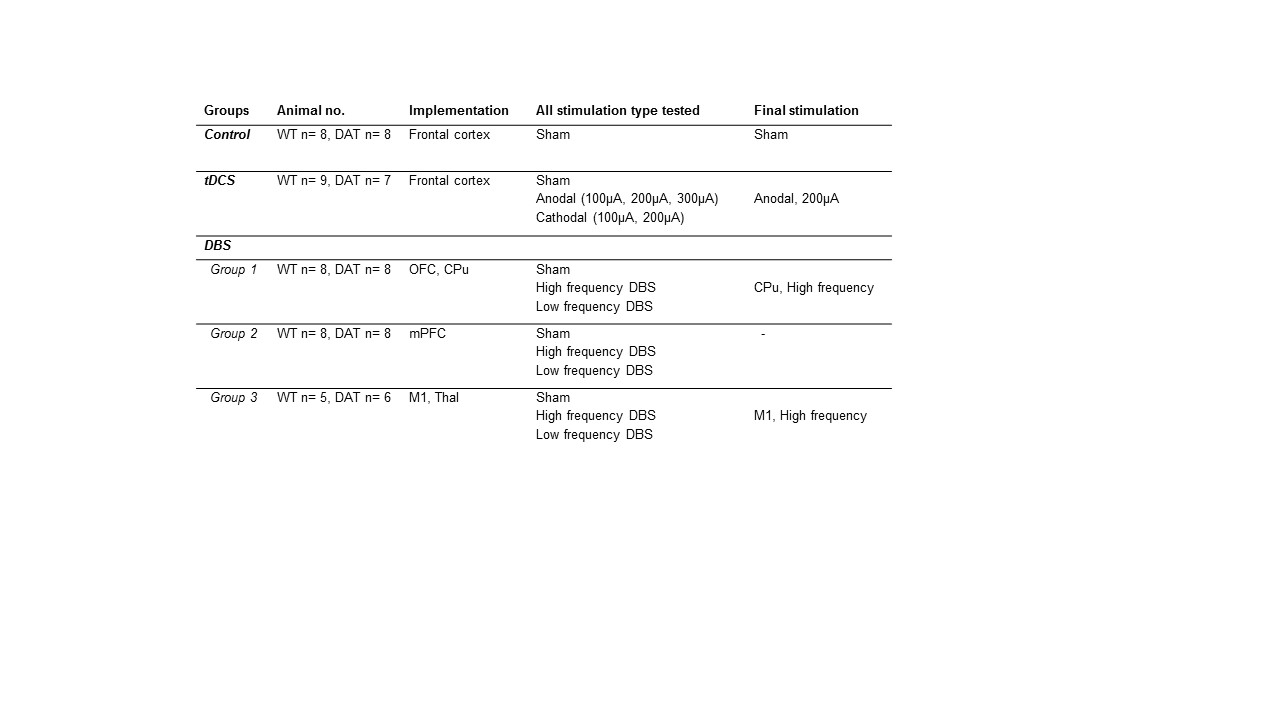


**Table S1: Group specifics.** Table showing the number of animals in the control, tDCS and DBS groups. For tDCS, stimulation was applied over the frontal cortex. Stimulation tested included sham, anodal (100µA, 200µA, 300µA) and cathodal (100µA, 200µA) stimulation. Either high or low frequency DBS was applied to the OFC and CPu (*group 1*), the mPFC (*group 2)* or the M1 and CmPf (*group 3).* For the final testing round, animals were stimulated with the most therapeutic-relevant setting as identified by the behavioral assessment. *wt*: wildtypes, *DAT-tg*: dopamine transporter overexpressing rats, tDCS: transcranial direct current stimulation, DBS: Deep brain stimulation, OFC: orbitofrontal cortex, CPu: caudate putamen, mPFC: medial prefrontal cortex, M1: primary motor cortex, Thal: thalamus.

1. **Neurobiological assessment**

**
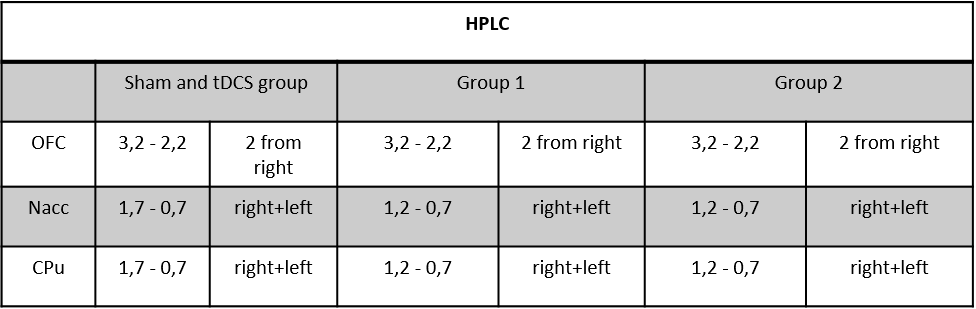
**

**
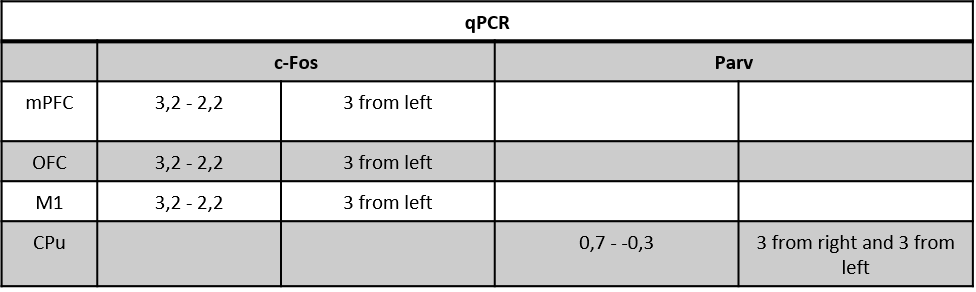
**

**Table S2: Neurobiological assessment.** Deep brain stimulation (DBS) animals that previously underwent behavioral scoring were subjected to DBS settings that elicited the most therapeutic behavioral results, i.e. high frequency DBS in either the caudate putamen (CPu) (group 1) or primary motor cortex (M1) (group 3) and sacrificed immediately thereafter. Brains were extracted and snap frozen for same post mortem assessment as animals in the transcranial direct current stimulation (tDCS) and sham groups. Table shows coordinates for coronal sections in accordance to bregma in mm anterior-posterior. For HPLC and qPCR quantification, animals from the following groups were used: sham, tDCS, group 1 (CPu) and group 3 (M1). Coordinates are in accordance to Paxinos & Watson brain atlas (1997). OFC: orbitofrontal cortex; NAcc: nucleus accumbens; mPFC: medial prefrontal cortex; Parv: parvalbumin

**
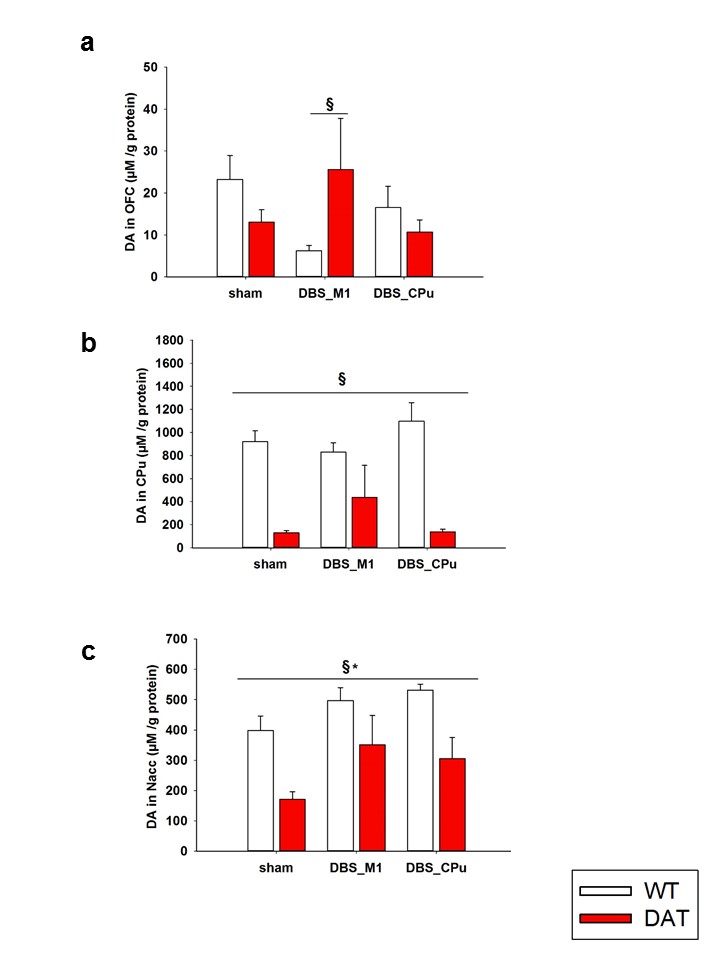
**

**Figure S2: Dopamine levels.** Figure showing dopamine content (µM/g protein) in the a) orbitofrontal cortex (OFC) b) caudate putamen (CPu) and c) nucleus accumbens (Nacc) following sham and high frequency deep brain stimulation (HF-DBS) to the primary motor cortex (M1) and CPu. In the OFC, a two-way ANOVA revealed a significant interaction (F(2,28)=4.807, p=0.016) with a further post-hoc showing significant difference in dopamine (DA) levels between sham and M1-DBS (a). In the CPu, a main effect was found for phenotype (F(1,29)=69,995 p<0.001) with dopamine transporter overexpressing rat *(DAT-tg)* rats displaying decreased DA levels in comparison to wildtype rats *(wt)* rats (b). In the Nacc, a significant main effect for phenotype (F(1,28)=19.329 p<0.001) and treatment (F(2,28)=4,825 p=0.016) with the lowest level of DA levels seen in the *DAT-tg* rats (c). All data are given as mean ± s.e.m. Asterisks (*) indicates significant difference between stimulation protocols, paragraph (§) indicates significant difference between phenotype with p < 0.05.

**
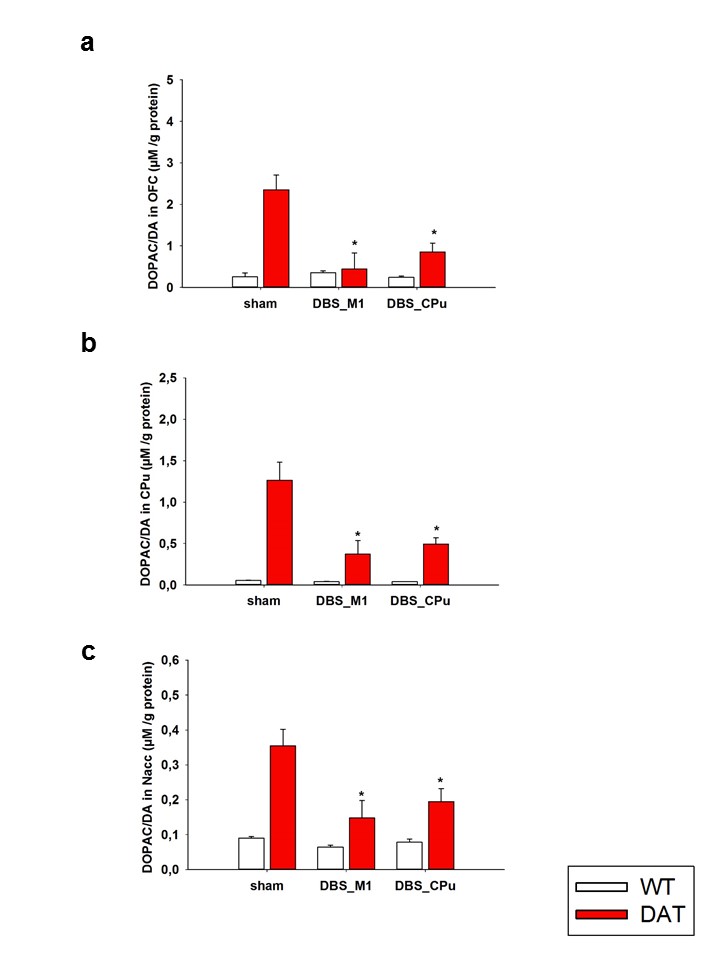
**

**Figure S3: Dopamine turnover.** Figure showing dopamine turnover (DOPAC/DA) (µM/g protein) in the a) orbitofrontal cortex (OFC) b) caudate putamen (CPu) and c) nucleus accumbens (Nacc) following sham and high frequency deep brain stimulation (HF-DBS) to the primary motor cortex (M1) and caudate putamen (CPu). In both the OFC, CPu and Nacc a significant main effect was found for phenotype (OFC, F(1,26)=16.837, p <0.001); CPu, F(1,29)=34.158 p<0.001; Nacc, F(1,27)=25.419 p<0.001), treatment (OFC, F(2,26)=7.302, p=0.003; CPu, F (2,29)=7.138 p=0.003; Nacc, F(2,27)=5.921 p=0.007) and interaction (OFC, F(2,26)=8.004, p=0.002; CPu, F(2,29)=6.708 p=0.004; Nacc, F (2,27)=3.841 p=0.034) with further post-hoc tests revealing significant differences in dopamine turnover following M1-DBS and CPu-DBS in the dopamine transporter overexpressing rat (DAT-tg) rats as compared to sham stimulation. All data are given as mean ± s.e.m. Asterisks (*) indicates significant difference between stimulation protocols, paragraph (§) indicates significant difference between phenotype with p < 0.05.

**
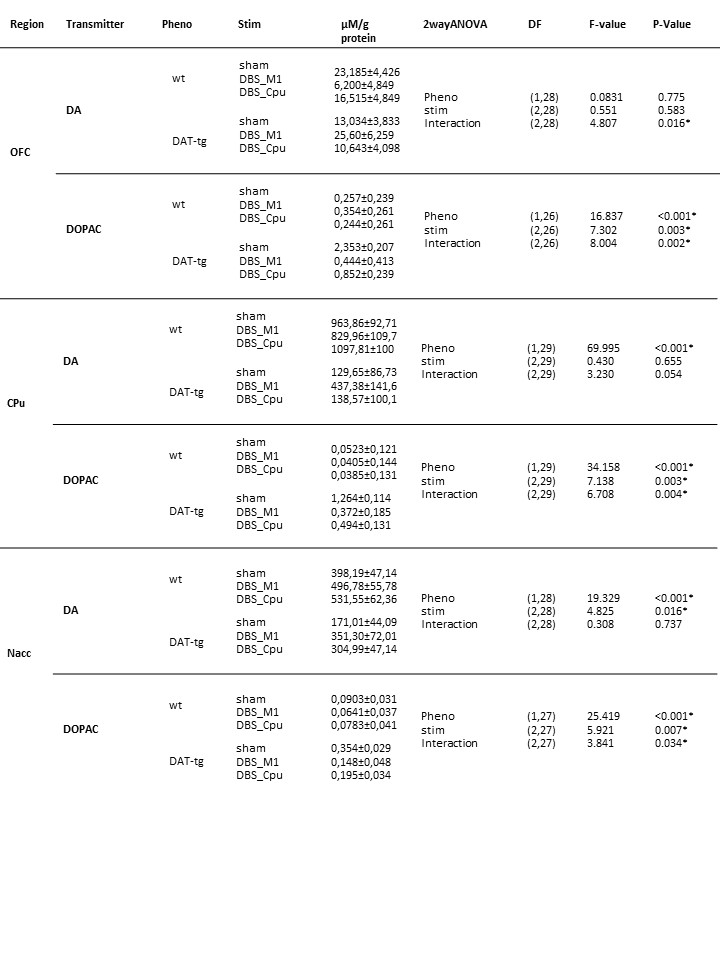
**

**Table S3: Neurotransmitter content.** Table summarizes neurochemical results. Neurochemical content was examined in the dopamine transporter overexpressing rat (*DAT-tg)* and wildetype (*wt)* rats following sham and deep brain stimulation (DBS) when applied to the caudate putamen (CPu) and primary motor cortex (M1). Dopamine levels and dopamine turnover were measured in the medial orbitofrontal cortex (OFC), nucleus accumbens (Nacc) and CPu. Data are presented as mean±s.e.m.

**
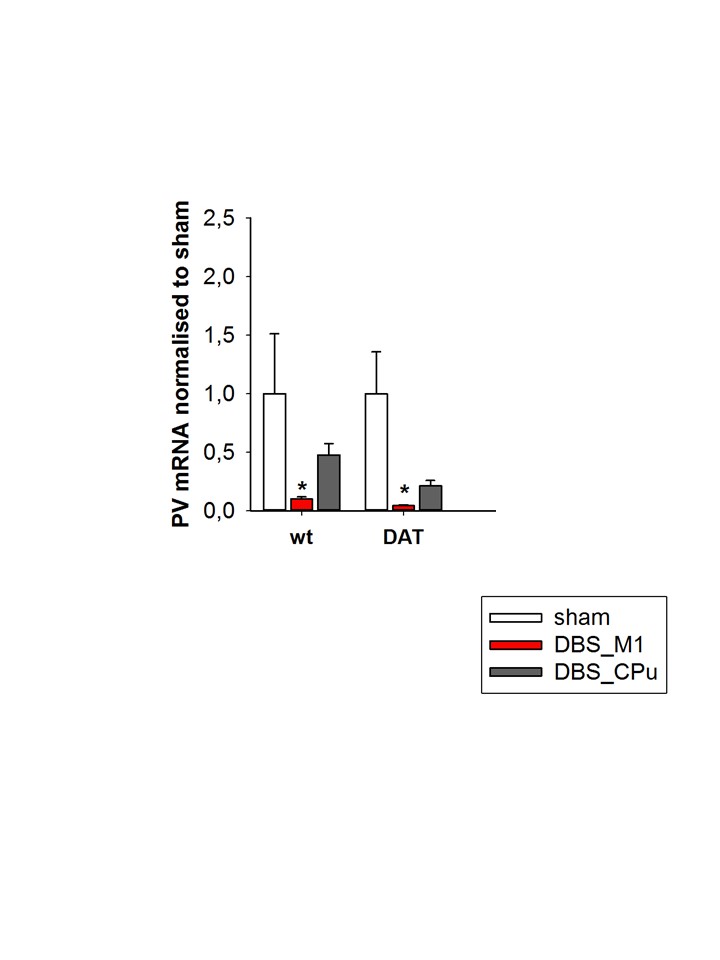
**

**Figure S4: Pv+ levels in the caudate putamen.** Figure showing the parvalbumin (Pv+) mRNA expression levels in caudate putamen (CPu) in the dopamine transporter overexpressing rat (*DAT-tg)* and wildetype (*wt)* rats following sham and deep brain stimulation (DBS) when applied to the CPu and primary motor cortex (M1). Following quantification of mRNA levels in the CPu, a Kruskal-Wallis One-Way analysis revealed a significant difference in PV+ expression levels between DBS-M1 relative to sham in both *DAT-tg* (H(2)= 9.692, p=0.008) and wt rats (H(2)= 9.038, p= 0.011). No significant difference was found following CPu-DBS relative to sham in either of the two phenotypes. All data are given as mean ± s.e.m. Asterisks (*) indicates significant difference between stimulation protocols, paragraph (§) indicates significant difference between phenotype with p < 0.05.

**
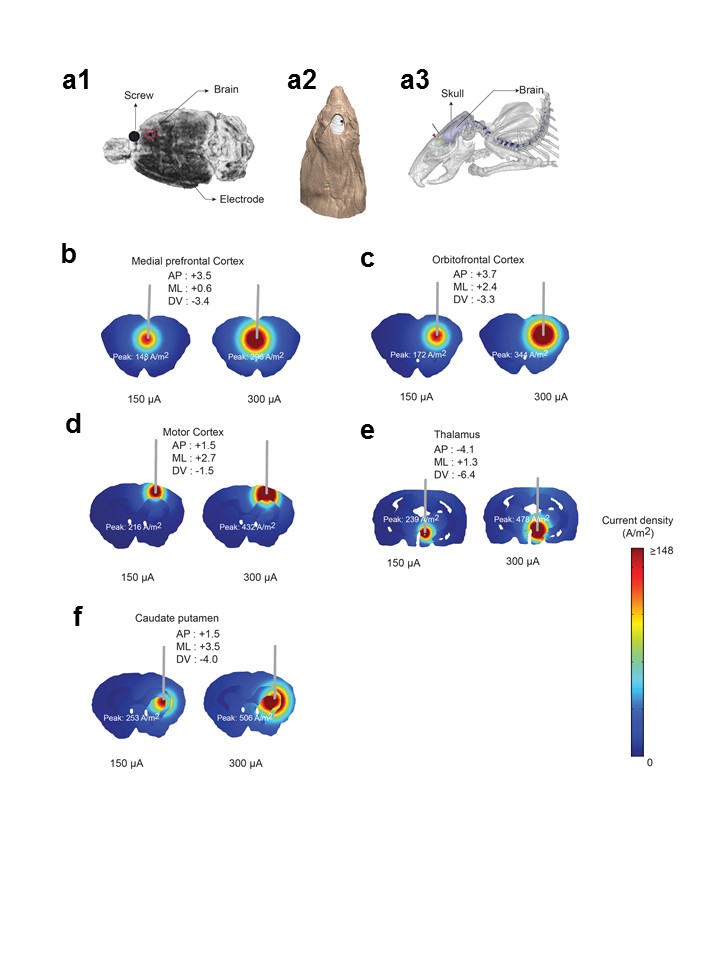
2. DBS modeling**

**Figure S5: DBS modeling.** Conductivity values were assigned as, scalp: 0.465 S/m; skull: 0.01 S/m; csf: 1.65 S/m; air: 1x10^-15^; gray matter: 0.276 S/m; cerebellum: 0.276 S/m; hippocampus: 0.126 S/m; white matter: 0.126 S/m; thalamus: 0.276 S/m, screw: 1.28 x10^8^; and electrode: 5.99 x 10^7^ S/m. (Bikson *et al.*, 2015; Song *et al.*, 2015) Computer aided model (CAD) geometry of the electrode and the screw were first imported into the head model and positioned for each montage (orbitofrontal cortex, medial prefrontal cortex, motor cortex, caudate putamen and thalamus) based on the coordinates values as used in the main manuscript. Volumetric meshes were later imported into COMSOL Multiphysics 4.3 (COMSOL Inc., MA, USA) to solve the model. The final finite element head assembly was solved for greater than 10,000,000 degrees of freedom and had greater than 8,500,000 tetrahedral elements. For electrical stimulation, a quasistatic approximation was implemented and boundary conditions were applied as normal current density (inward current flow) at the exposed surface of the electrode and ground at the surface of the screw. Remaining external surfaces of the model were electrically insulated. Simulation was carried out for both 150 µA and 300 µA current intensities. Current density slice plots for each intensities and montages were generated and the peak values were reported. The predicted current density plots for high- and low frequency DBS application and corresponding was assessed through computational modeling. Electrode and a screw configuration in one of the montage is shown as an exemplary configuration of electrode positioning at the incision area (a). Current density for each electrode configuration was doubled when intensity was increased by two-fold. On a cortical level, maximum current density was observed in the M1 following HF-DBS (300 µA = 432 A/m^2^). On a subcortical level, maximum current density was found in the CPu following HF-DBS (300 µA = 506 A/m^2^) (b-f).
